# Dataset of solution-based inorganic materials synthesis procedures extracted from the scientific literature

**DOI:** 10.1038/s41597-022-01317-2

**Published:** 2022-05-25

**Authors:** Zheren Wang, Olga Kononova, Kevin Cruse, Tanjin He, Haoyan Huo, Yuxing Fei, Yan Zeng, Yingzhi Sun, Zijian Cai, Wenhao Sun, Gerbrand Ceder

**Affiliations:** 1 Department of Materials Science and Engineering, University of California, Berkeley, CA 94720 USA; 2 Materials Sciences Division, Lawrence Berkeley National Laboratory, Berkeley, CA 94720 USA; 3 Department of Materials Science and Engineering, University of Michigan, Ann Arbor, MI USA

**Keywords:** Materials science, Computational methods, Cheminformatics, Design, synthesis and processing

## Abstract

The development of a materials synthesis route is usually based on heuristics and experience. A possible new approach would be to apply data-driven approaches to learn the patterns of synthesis from past experience and use them to predict the syntheses of novel materials. However, this route is impeded by the lack of a large-scale database of synthesis formulations. In this work, we applied advanced machine learning and natural language processing techniques to construct a dataset of 35,675 solution-based synthesis procedures extracted from the scientific literature. Each procedure contains essential synthesis information including the precursors and target materials, their quantities, and the synthesis actions and corresponding attributes. Every procedure is also augmented with the reaction formula. Through this work, we are making freely available the first large dataset of solution-based inorganic materials synthesis procedures.

## Abstract (summary)

**Table:** 

Measurement(s)	solution-based inorganic synthesis data
Technology Type(s)	natural language processing

## Background & Summary

Big-data-driven approaches have helped to establish a new paradigm of scientific research^1–3^. In materials science specifically, the Materials Genome Initiative (MGI) effort has significantly facilitated and accelerated materials discovery and design by deploying large-scale *ab initio* computation and building computed databases of structure–property relationships^4–6^. Unlike computational data, the experimentally determined properties and structures of inorganic materials are mainly available in manually curated databases^7–11^. Such well-curated experimental databases have led to early machine learning models that address difficult problems in materials research, such as structure prediction^3^. The ability to efficiently design and predict the structure of novel advanced materials with the assistance of computed and experimental databases has shifted the materials innovation challenge toward understanding and determining the synthesis routes for novel materials^12^. In principle, this challenge could also be mastered using data-driven approaches, since only limited predictive theories are available for materials synthesis^13–23^. Indeed, in organic chemistry, AI-guided synthesis planning^24,25^ has already been successfully implemented in certain cases, such as in predicting retrosynthesis^26^ and in complex natural product synthesis design^27^. Although datasets for the synthesis of organic materials are widely available^28,29^, there is not yet a large-scale database of inorganic synthesis routes, which is needed to train advanced deep-learning models to enable a breakthrough in AI-assisted design and optimization of inorganic materials synthesis.

Scientific publications represent the largest repository of knowledge about materials synthesis and can be used as a reliable source of data. However, human-written descriptions of syntheses require additional levels of interpretation for conversion into a codified, machine-operable format. Additionally, manual extraction of synthesis information is laborious, even for a very limited number of papers^30–33^. Given these obstacles, an automated information extraction pipeline can accelerate data collection and assist in building structured synthesis procedures from scientific text. Natural language processing (NLP) approaches have been widely developed in the past decade, and various advanced tools for high-quality information extraction from unstructured text are available to researchers.

In materials science, NLP has been used to extract and analyze materials properties^34–36^, applications^37,38^, and synthesis conditions for some limited cases^39^. Various NLP tools, including ChemDataExtractor^40^, OSCAR^41^, ChemicalTagger^42^, and others^30,43,44^, have been developed to extract information from chemical text. Recently, advanced models based on deep convolutional and recurrent neural networks^45–48^ have been proposed to improve the accuracy of chemical data extraction.

Text-mining approaches in materials science have also been used to construct automated pipelines for collecting information about materials synthesis from publications and to build large-scale publicly available datasets from such collected data, including datasets of synthesis formulations for metal oxides^39,49,50^, germanium-containing zeolites^51^, and perovskites^52^. In recent work, our group has developed a text-mining pipeline to construct the first large-scale dataset of solid-state ceramics synthesis “recipes”, which includes not only the starting materials and final products but also the synthesis actions, their attributes, and balanced chemical-reaction equations^53^.

**Fig. 1:**
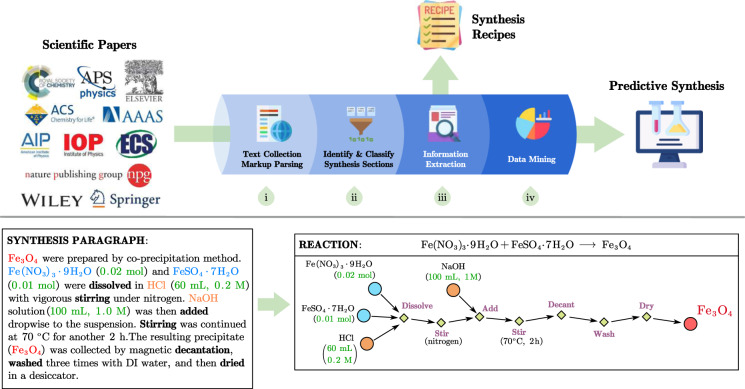
Extraction pipeline and example. *Top panel:* Schematic representation of the standard text mining pipeline: (i) scrape papers in markup format from the major publishers; (ii) identify and classify synthesis sections; (iii) extract key information including materials, amounts, sequenced operations, and conditions; (iv) store synthesis procedures into the database for future data mining. *Bottom panel:* Example of a codified procedure extracted from a synthesis paragraph.

In the current work, we built a more advanced extraction pipeline (Fig. 1) which uses various advanced machine learning and natural language processing techniques to extract precise data for solution-based inorganic materials synthesis procedures from the scientific literature. Solution procedures are considerably more complex than solid state synthesis and require the precise extraction of not only the chemicals involved but also their respective amounts (since they determine concentration in solution). In addition, more complex organic and mixed organic-inorganic compounds are used to solubilize ions or to control solution conditions. By applying the extraction pipeline, we codified 35,675 solution-based inorganic materials synthesis procedures from over 4 million papers. Extracted information includes target material and precursors, their quantities, and the synthesis operations and their attributes. Information about the targets and precursors is then used to build a reaction formula for every synthesis procedure. This dataset is the first large-scale dataset of solution-based synthesis procedures, and provides a foundation to test and verify existing empirical synthesis rules, improve prediction accuracy, and even data-mine new rules to guide synthesis. Also, this codified dataset should pave the way to design optimized synthesis procedures in automated experimentation.

## Methods

### Content acquisition

The journal articles used in this work were downloaded with publisher consent from Wiley, Elsevier, the Royal Society of Chemistry, the Electrochemical Society, the American Chemical Society, the American Physical Society, the American Institute of Physics, and Nature Publishing Group. A customized web-scraper, Borges (see Codes Availability section below), was used to automatically download a broad selection of materials-relevant papers published after the year 2000 from publishers’ websites in HTML/XML format. We selected 2000 as the cutoff year as parsing of materials science papers stored as image PDFs (as for most papers published before 2000) introduces a significant number of errors due to the limitations of currently available optical character recognition models on chemistry-containing text^54,55^.

To convert the articles from HTML/XML into raw-text files, we developed the LimeSoup toolkit (see Codes Availability section below), which takes into account the specific format standards of various publishers and journals. The full-text and metadata of the articles such as the journal name, article title, abstract, author names, etc., are stored in a MongoDB (www.mongodb.com) database collection. To date, we have accumulated 4.06 million articles, which are used for further processing down the pipeline (Fig. 1).

### Paragraph classification

Paragraphs containing information about solution synthesis (referred to as “synthesis paragraphs” throughout this paper) were identified using a Bidirectional Encoder Representations from Transformers (BERT) model^56^. The model was pre-trained on full-text paragraphs of 2 million papers randomly drawn from our database in a self-supervised way, i.e., by predicting masked words based on their surrounding context. After training the BERT model, we fine-tuned the paragraph classifier using 7,292 paragraphs labeled as either “solid-state synthesis”, “sol-gel precursor synthesis”, “hydrothermal synthesis”, “precipitation synthesis”, or “none of the above”. The resulting F1 score of the paragraph classification is 99.5%, an improvement over the F1 score of 94.6% in our previous work^57^, when evaluated using the same labeled training dataset.

### Synthesis procedure extraction

A solution-based synthesis procedure includes the precursors and target materials, their quantities, and the synthesis actions and their attributes, properly sequenced. This is the minimum essential information required to complete a synthesis route. A schematic representation of the procedure is shown in the bottom panel in Fig. 1. In the sections below, we provide a brief overview of the methods used for each step of the procedure extraction.

### Materials entity recognition (MER)

Materials entities in synthesis paragraphs are identified and classified as *target*, *precursor*, or *other* via a two-step sequence-to-sequence model as introduced in our previous work^46^. In the current work, we replaced the original Word2Vec embedding model used previously^58^ with a BERT model trained on papers from the materials science domain (see Section “Paragraph classification” above). First, each word token was transformed into a digitized BERT embedding vector. A bi-directional long-short-term memory neural network with a conditional random-field top layer (BiLSTM-CRF) was used to determine whether the token was a materials entity or a regular word, and each materials entity was replaced with the keyword <MAT> before being classified as either a *target*, *precursor*, or *other* material using a second BERT-based BiLSTM-CRF network. In addition to the 834 annotated solid-state synthesis paragraphs from 750 papers used in our previous work^46^, we manually annotated 447 solution-based synthesis paragraphs from 405 papers by labeling each word token as *material*, *target*, *precursor*, or *outside*. The annotated dataset was split into training, validation, and test sets with a paper-wise ratio of 700:150:305 to train the aforementioned two neural networks.

### Extraction of synthesis actions and attributes

We implemented an algorithm which combines a neural network and sentence dependency tree analysis to identify synthesis actions in the text. First, the Word2Vec model from the Gensim library^59^ was re-trained on ~400,000 synthesis paragraphs of four synthesis types (see Section “Paragraph classification” above). These word embeddings were used as the input for a recurrent neural network that takes a sentence word-by-word and assigns labels to the verb tokens: *not-operation*, *mixing*, *heating*, *cooling*, *shaping*, *drying*, or *purifying*. For each obtained synthesis action, we parsed a dependency sub-tree using the SpaCy library^60^ to obtain information about the corresponding temperature, time, and environment. To extract the corresponding values of these attributes, we used a rule-based regular expression approach^61^.

### Extraction of material quantities

To extract the numerical values of material quantities and assign them to the corresponding materials obtained using the MER model (see Section “MER” above), we applied a rule-based approach to search along the syntax tree^61^. The NLTK library^62^ was used to build the syntax trees for each sentence in a paragraph. The words in given sentences are leaf nodes of syntax trees. We then applied an algorithm to cut the syntax tree of each sentence into the largest sub-trees for every material, with each sub-tree having only one material entity: 1. we first identified the materials on leaf nodes; 2. starting from each material, we identified the largest sub-trees, i.e., we traversed the syntax tree upwards until there was more than one material leaf node descending from the same node; 3. the largest sub-tree for a given material was defined as the sub-tree formed by the node and its descendants identified in step 2. Next, we searched for the quantities in each sub-tree given as molarity, concentration, or volume. Finally, we assigned the quantities found to the unique material entity in the sub-tree.

### Building reaction formulas

For every synthesis procedure described in a paragraph, we built a chemical formula. Every material entity was converted from a text-string representation into a chemical-data structure using an in-house material parser toolkit (see Codes Availability section below). The data structure included information about the material formula, composition, and ions. We then paired the target with precursors containing at least one element in the target except for hydrogen and oxygen and defined those precursors as “precursor candidates”. Next, we computed the oxidation state change of elements from each “precursor candidate” to the target and determined whether the precursor was oxidized or reduced. If precursors were reduced or oxidized, we also included the corresponding redox agents in the reaction formula. The agents can either be another “precursor candidate” or a commonly used oxidizing or reducing agent from the remaining material entities marked as *other* or *precursor* by the MER algorithm (see Section “MER” above).

### Dataset generation

The dataset generation followed the protocol displayed in Fig. 1. We downloaded a total of 4,061,814 papers using web scraping and identified the experimental sections by keyword matching in section headings, with keywords including “experiment”, “synthesis”, “preparation”, and their morphological derivations. ChemDataExtractor^40^ was used to split the plain-text paragraphs into sentences and words. After classification (see Section “Paragraph classification” above), 364,076 paragraphs describing solid-state, hydrothermal, sol-gel, and precipitation syntheses were obtained. Among them, 189,553 paragraphs described hydrothermal or precipitation syntheses, which we categorize as solution-based synthesis methods. These paragraphs were further processed to extract the precursors, targets, quantities, and operations with corresponding conditions and to build the reaction formula (Fig. 1).

## Data Records

The solution-based synthesis dataset is provided as a single JSON file, available at 10.6084/m9.figshare.16583387.v4^63^. There are 20,037 hydrothermal synthesis reactions and 15,638 precipitation synthesis reactions. Each record corresponds to a synthesis procedure extracted from a paragraph and is represented as an individual JSON object. If a paragraph reported the synthesis of several materials, the corresponding reactions were split into separate data records. In addition to the chemical formula, the metadata for each reaction returns the data structure used in our previous work^53^, which includes: DOI of the paper, a snippet of the corresponding synthesis paragraph (50 first and 50 last characters to facilitate its lookup), chemical information about the target and precursor materials used in the reaction, and operations with their corresponding attributes. We also included the materials with their corresponding quantities in the metadata. The details of the data format are given in Table 1

**Table 1:** Format of each data record: description, key label, data type.

Data description	Data Key Label	Data Type
DOI of the original paper	doi	*string*
Snippet of the raw text	paragraph_string	*string*
Chemical formula	reaction	Object *(dict)*:
		- left_side: *list of strings*
		- right_side: *list of strings*
Chemical formula in string format	reaction_string	*string*
Target material data	target	Object *(dict)*:
		- material_string: *string,*
		- material_formula: *string,*
		- composition: *list* of Objects1,
		- additives: *list* of strings
		- elements_vars: {var: *list* of *strings*}
		- amounts_vars: {var: *list* of Objects2}
		- oxygen_deficiency: *boolean*
		- mp_id: *string*
List of target formulas obtained after variables substitution	targets_string	list of *strings*
Precursor materials data	precursors	*list* of Objects (See target)
List of solvent formulas	solvents_string	*list* of *strings*
Sequence of synthesis steps and corresponding conditions	operations	*list* of Objects (*dict*):
		- token: string,
		- type: *string*
		- conditions: Object
		–temperature: *list* of Objects3
		–time: *list* of Objects3,
		–atmosphere: *list* of *strings*
		–mixing_device: *list* of *strings*
		–mixing_media: *list* of *strings*
Materials with corresponding quantities	quantities	*list* of Objects (dict):
		- material: *string*,
		- quantity: *list* of Objects4
Synthesis type	type	*string*

^1^{*formula*: *string*, *elements*: {*elements*: amount of element}, amount: *string*}.

^2^{*max_value*: *float*, min_value: *float*, *values*: *list of floats*}.

^3^{*max_value*: *float*, *min_value*: *float*, *values*: *list* of *floats*, *units*: *string*}.

^4^{*number*: *float*, *unit*: *string*}.

The chemical formula for the reaction is stored as a string (reaction_string) as well as in a dictionary containing lists of precursors (left_side) and target materials (right_side) in the reaction.

The metadata for target materials and precursors used to construct the chemical formula are represented by the following data structure:

- material_string: string of material as given in the original paragraph before being parsed into a chemical composition.
- material_formula: chemical formula associated with the material (given originally or constructed empirically by parser).
- composition: chemical composition of the material derived from its formula. Aside from single-compound materials, we found that a large portion of the materials (predominantly target materials) are composites, mixtures, solid solutions, or alloys written as a sequence of compound-fraction pairs. Therefore, a chemical-composition entity is represented by a list of dictionary entries, where each item is associated with a compound found in the materials formula. The fraction of each compound in the material is given in amount, and its chemical composition (i.e., the elements and stoichiometry) is given in elements. If a material is one compound, the list has only one item and amount = 1.0. If a material is a hydrate, water is added to the composition list with its amount corresponding to the amount of water molecules (if specified).
- additives: list of additive elements (i.e., elements used for doping, stabilization, or substitution) resolved from the material string.
- elements_vars: lists all variable elements and their corresponding values found in the materials.
- amounts_vars: lists all variable element ratios and their corresponding values found in the material formula. The values of each variable are given as a structure with values listing the values of each specific variable and max_value/min_value values if a range is given in the paragraph.
- oxygen_deficiency: yes/no attribute that reflects whether a material was synthesized with unspecified oxygen stoichiometry.
- mp_id: ID of the lowest-energy polymorph entry in the Materials Project database (https://materialsproject.org/materialsproject.org) if the material is found there.

To facilitate querying of the dataset, the targets_string field contains the target material formulas, and the solvents field contains all solvent(s) from matching material entities marked as *other* by the MER model with a table of common solvents adopted from Common Solvents Used in Organic Chemistry (https://organicchemistrydata.org/solvents/organicchemistrydata.org/solvents).

## Technical Validation

### Extraction completeness and accuracy

To ensure high accuracy of the dataset, we included only those data that produced complete reaction formulas at the final step of the pipeline. This strategy reduced potential errors in the dataset that may have been caused by composition-parsing failure, incomplete extraction, or incomplete information provided by the text. We applied the extraction pipeline to 189,553 solution-based synthesis paragraphs, 28,749 of which generated a reaction formula, giving an extraction yield of ~15%. To evaluate the source of the loss, we randomly selected and manually checked 100 solution-based synthesis paragraphs that did not produce any reactions. Among those 100 paragraphs, 36 were written with an incomplete list of precursors or targets in the text, such that human experts would not be able to reconstruct the reaction based solely on the information provided in the paragraph. For the remaining 64 paragraphs, the loss was due to: 1. the use of organic precursors with complex groups or complicated notation (e.g., acronyms) that could not be parsed into a chemical composition by our parser or 2. MER misidentification resulting in an incomplete or incorrect list of precursors and (or) target entities such that the reactions could not be built.

To evaluate the quality of the dataset, we had a human expert test 100 randomly pulled entries. The human expert manually extracted the information presented in the procedure, and the results were compared with those extracted by the pipeline. Table 2 presents the accuracy statistics, which include the precision, recall, and F1 scores calculated from the tested entries. For the fields that included reaction, targets, precursors, operations, operation temperatures, time, and atmosphere, the F1 scores were over 90%. The relatively low recall, and hence F1 score, for the extraction of materials quantities can be mainly explained by the MER algorithm missing the corresponding material entity and, thus, the quantities not being assigned. The accuracy of the obtained dataset is comparable to that in our previous work^53^ and of other text-mined datasets^49^.

**Table 2:** Performance of data extraction for dataset entries.

Data attribute	Precision	Recall	F1 score
Balanced reactions	0.94	/	/
- targets	0.97	/	/
- precursors	0.98	0.99	0.98
Operations	0.96	0.85	0.90
Conditions			
- temperature	0.97	0.92	0.94
- time	0.98	0.89	0.93
- atmosphere	0.97	0.92	0.94
Quantities	0.90	0.85	0.87

### Exploratory data analysis

To test the diversity of the dataset and its coverage of the materials space, we analyzed unique materials (targets and precursors) and reactions. The dataset contains 11,603 unique reactions that include 2,870 unique precursors and 5,416 unique targets. The ten most frequent targets in the dataset and their corresponding precursors are listed in Table 3. The target list captures materials that have drawn substantial attention in the past two decades: catalysts (ZnO, Fe_2_O_3_, TiO_2_, Fe_3_O_4_, SnO_2_, ZrO_2_, CuO), adsorbents (SiO_2_), various materials for sensors (ZnO, Fe_2_O_3_, WO_3_), quantum dots (CdS), and semiconductors (ZnO, TiO_2_, SnO_2_, CdS). Unsurprisingly, these most frequent target materials usually appear in multiple applications, as they possess desirable physical and chemical properties in many scientific and engineering fields.

**Table 3:** Ten most common targets in the dataset with their corresponding precursors.

Targets	Common Precursors
ZnO	Zn(NO_3_)_2_, Zn(Ac)_2_, ZnCl2
TiO_2_	Ti(OCH(CH_3_))_4_, Ti(OC_4_H_9_)_4_, TiCl_4_
Fe_3_O_4_	FeCl_3_, FeCl_2_
Fe_2_O_3_	FeCl_3_, Fe(NO_3_)_3_
SnO_2_	SnCl_4_
ZrO_2_	ZrOCl_2_, ZrO(NO_3_)_2_
CuO	Cu(NO_3_)_2_, Cu(Ac)_2_, CuCl_2_, CuSO_4_
SiO_2_	Si(OC_2_H_5_)_4_
WO_3_	Na_2_WO_4_, WCl_6_, H2WO_4_
CdS	Na_2_S, CH_4_N_2_S, CdCl_2_, Cd(NO_3_)_2_

**Fig. 2:**
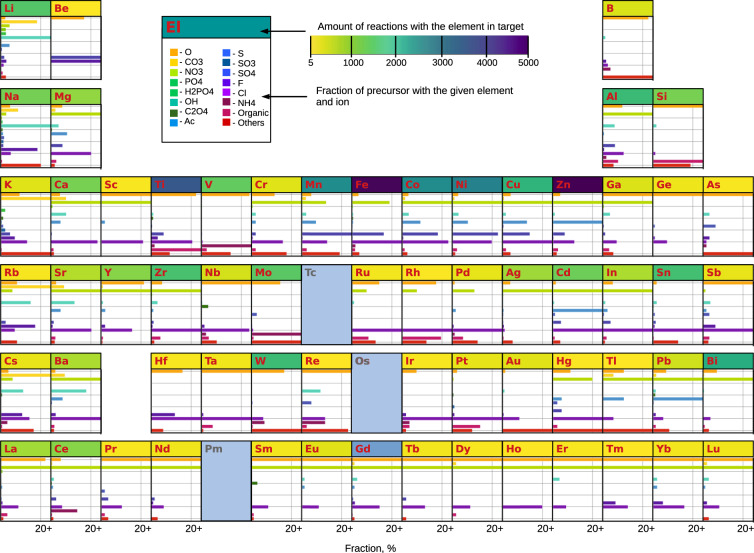
The chemical space covered by the dataset. For each element, the box containing the element name is colored in a yellow-to-navy blue gradient representing the total amount of reactions that produce a target compound containing the element. The bar graph below each element shows the list of ions paired with the element in precursor compounds. The fractions of the precursors (i.e. element + ion) used are shown by the length of the bars. Boxes with no bar graph represent elements occurring in five and fewer targets. “Ac” stands for acetate radical CH_3_COO^−^ in the compound formula.

We use the periodic table representation (Fig. 2) to visualize the chemical space covered by the dataset. For each element, the fraction of synthesis procedures containing this element in the target formula is shown with the yellow-to-navy blue gradient framed at the top of each element box. The most data-rich elements are transition metals in the third period, such as Zn, Fe, Ti, Ni, and Co, in accordance with the compounds listed in Table 3. The next-most prevalent targets are materials with Bi, Sn, Al, W, Mo, Cu, Zr, or Li. The least common elements are rare elements such as Ru, Rh, Hf, Ta, Re, and Ir. The elements Fr, Ra, Tc, and Pm are not present as target materials in the dataset, likely due to their radioactivity. Additionally, we calculated the frequency of co-occurrence of chemical elements and common ions in precursor materials to understand how different ions are brought into solution. In Fig. 2, the frequencies for each ion are displayed as colored bars. The length of the bar is the fraction of one specific ion paired with the element normalized over all precursors for this element.

The commonly used precursors are mainly those that are widely available from companies such as Sigma-Aldrich and Fisher Scientific. For example, Li_2_CO_3_ or LiOH for Li and sulfate or chloride for Fe. Inorganic salts, such as nitrates, sulfates, and chlorides, are often used because of their high solubility^64^. Halides are not used for Lanthanide metals because Lanthanide halides are highly hygroscopic, and thus their molar weight is not well defined. We observed that precursors with neighboring elements in the periodic table tend to have similar ions paired. For instance, nitrates, sulfates, and chlorides are commonly used anions for 3rd-period transition metals, whereas the precursors for lanthanides are mostly oxides and nitrates.

**Fig. 3:**
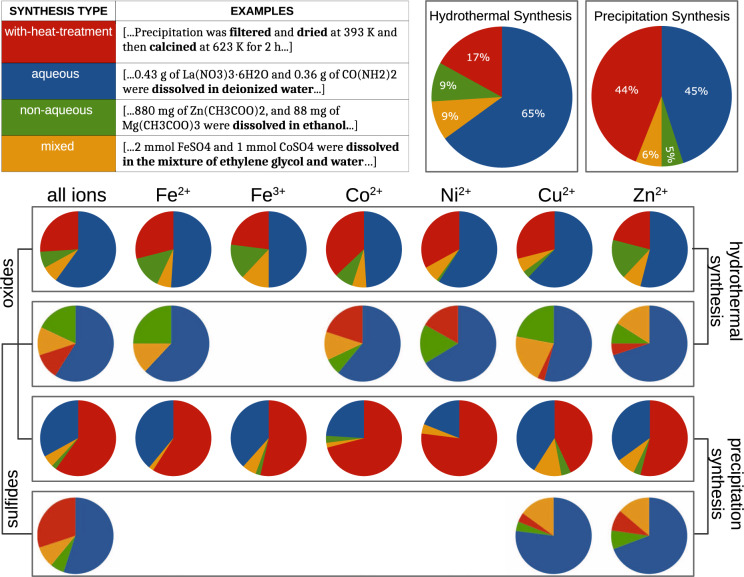
Correspondence between choice of synthesis route and selected types of targets. The top table gives an example of the four synthesis categories defined: with heat treatment step, aqueous, non-aqueous, and mixed. The two pie-charts on the top-right show the fractions of synthesis routes in the hydrothermal and precipitation datasets separately. The four rows of pie charts in the lower half of the figure represent the fractions of the four synthesis routes (given in the table) for all oxides, all sulfides, and individual oxides and sulfides with different oxidation states of data-rich transition metals separately. The first and second rows are results from the hydrothermal dataset. The third and fourth rows are results from the precipitation dataset. Each blank space means that there is not enough data to form a statistic for the corresponding type of target.

We used information about the extracted materials and sequences of synthesis actions to classify the solution-based synthesis procedures into four categories of synthesis protocols (table in Fig. 3) according to the following definitions:

- *solution-mixing with heat treatment step* has a final heat treatment step after the precipitate is obtained from the solution;
- *aqueous solution synthesis* has no final heat treatment step after precipitating the compound from the solution and the solvent is water;
- *non-aqueous solution synthesis* has no final heat treatment step after precipitating the compound from solution and the solvent(s) is (are) organic;
- *aqueous–non-aqueous mixed solution synthesis* has no final heat treatment step after precipitating the compound from solution and the solvents are a mixture of water and organic solvent(s).

The resulting distributions of synthesis protocols over the aforementioned categories are shown in the two pie charts in the top-right corner of Fig. 3. Note that as solution-based synthesis includes both hydrothermal and precipitation synthesis according to our definition (see Section “Paragraph classification”), we analyzed these synthesis types separately. As observed in the pie charts, only 20% of the procedures in the hydrothermal synthesis subset have a heat treatment step after solution mixing. Among those that do not have heat treatment step, 63% use only water as a solvent, 8% use only organic solvents, and 9% use both water and organic solvents. In contrast, the fractions in the precipitation synthesis subset are 43%, 46%, 5%, and 6%, respectively.

A heat treatment step after solution mixing can be used to dehydrate the targets, decompose the intermediates to produce the final products, change the oxidation state, change the morphology, or improve crystallization^65–67^. To explore this in more detail, we split the targets according to their anion type (oxide, sulfide, etc.) and different oxidation states of several data-rich transition-metal elements. We then computed the distribution of synthesis categories for each of the split subsets. Figure 3 presents the results for the most prevalent subsets of oxides, sulfides, and elements Fe^2+^, Fe^3+^, Co^3+^, Ni^2+^, Cu^2+^, and Zn^2+^. The fraction of procedures with a heat treatment step in precipitation synthesis is larger than that in hydrothermal synthesis. This observation holds for all targets, all oxides, all sulfides, and individual oxides and sulfides with queried oxidation states of transition metals. This finding can also be interpreted as hydrothermal synthesis often being used to obtain final products in a “one-shot” process, without subsequent heat treatment after solution mixing, likely because many compounds can be crystallized as anhydrous powders with controlled size and morphology directly from hydrothermal synthesis. In a standard hydrothermal synthesis procedure, the reaction is performed in an autoclave with autogenic pressure so that it can operate in a wider temperature window, including temperatures above the atmospheric boiling point of the solvent. In contrast, precipitation synthesis is performed under normal pressure. The higher temperature possible in hydrothermal synthesis is associated with enhanced kinetics in chemical transport, nucleation, and crystal growth and thus with a more effective dissolution–recrystallization process, which can help remove defects and improve crystallinity. Furthermore, the physico-chemical properties, such as the viscosity and dielectric constant of water or other solvents, change pronouncedly under conditions of hydrothermal synthesis, affecting the solubility and mobility of species in the solution and eventually facilitating crystallization^68^. Therefore, hydrothermal synthesis does not need a post-synthesis heat treatment as often as precipitation synthesis.

Solution-based synthesis is an important area of materials synthesis^57^ and this dataset can help with advancing the science and model building for solution synthesis. Nevertheless, challenges remain in the mining of scientific literature and construction of robust and accurate large-scale datasets. First, the organic precursors with complex radicals commonly used in solution-based synthesis pose a challenge for parsing and extracting chemical information. Constructing reaction formulas becomes problematic when the precursor information is lost. Therefore, these entries are mostly dropped out later in the pipeline. To address this issue, a universal parser that can parse chemical tokens needs to be developed.

Second, our data was extracted from the experimental section in the main body of each paper and does not include any information about the actual synthesis results, e.g., whether the material was synthesized using the reported procedure or which structure was obtained. This problem could be overcome by introducing a model that can parse characterization data (e.g., X-ray diffraction patterns or electron microscopy images) and relate them to the corresponding synthesis conditions, something which, to the best of our knowledge, has not yet been performed. Even though the actual results of a synthesis can be extracted from a paper, there remains the challenge of data interpretation and usage, as the authors usually report only successful and “cherry-picked” experimental results. This introduces significant anthropogenic bias toward “positive” data with little “negative” content in the dataset, thus limiting the tasks for future machine-learning applications^69,70^. A promising approach to solve this issue is to incorporate results obtained by autonomous robotic synthesis platforms that can provide a vast amount of “negative” data in a reasonable time frame^71,72^.

Finally, solution-based synthesis is advantageous when the control of specimen morphology is required, e.g., when synthesizing noble-metal nanoparticles. However, this dataset does not provide information about the morphology of the synthesized materials, though such information is often contained in characterization or results paragraphs instead of the experimental section. The extraction of morphology and other solution synthesis outcomes is another text-mining challenge in materials science research that requires the development of advanced algorithms and models^30^, which is beyond the scope of the current study.

## Usage Notes

The dataset is provided in JSON format as a single file. All major programming languages, such as Python, Matlab, R, and Wolfram Mathematica, can be used to read it. No particular dependency is required.

Because the dataset contains detailed information about chemical formulas as well as the compositions of the target materials and precursors for each procedure, it can be easily used to conduct a literature review by querying desired precursors and (or) targets in different chemical spaces. For example, selecting all TiO_2_ synthesized from TiCl_4_ allows an exploration of how other synthesis formulations, such as synthesis actions, attributes, and quantities, affect the results. Furthermore, the materials entries in the dataset are supplied with the Materials Project^5^ identifiers, thus facilitating the integration of the procedures with the thermochemical data available in the Materials Project^73,74^.

In addition, this solution-based synthesis dataset keeps the same data structure as that in the solid-state dataset generated in our previous work^53^. Therefore, it is easy to analyze the procedures from the two datasets.

Despite the dataset being provided as a static snapshot^63^, we intend to update it on a regular basis.

## Data Availability

The scripts used to classify paragraphs and extract procedures as well as to perform the data analysis are home-written codes which are publicly available at the GitHub repository https://github.com/CederGroupHub/text-mined-solution-synthesis_public with acknowledgement of the current paper. The underlying libraries used in this project are all open-source: *Tensorflow* (www.tensorflow.org) *Keras* (keras.iokeras.io) *SpaCy* (spacy.iospacy.io)^60^ *NLTK* (https://www.nltk.org/)^62^ *gensim* (radimrehurek.comradimrehurek.com)^59^ *scikit-learn* (scikit-learn.org)^75^ *ChemDataExtractor* (chemdataextractor.org)^40^ *Material Parser* (github.com/CederGroupHub/MaterialParser) *Borges* (github.com/CederGroupHub/Borges) LimeSoup (github.com/CederGroupHub/LimeSoup).
